# Acknowledgement

**DOI:** 10.3402/gha.v9.31281

**Published:** 2016-03-09

Scientific journals depend on committed and qualified peer reviewers when it comes to publishing scientifically rigorous and high-quality content. In recognition of this essential role, *Global Health Action* would like to thank all peer reviewers that completed reviews in 2015 (listed below) – your voluntary contribution and commitment is greatly appreciated.

We look forward to continuing our collaboration in 2016 - and welcome you as authors as well.

Abdullah, Asnawi

Abimanyi-Ochom, Julie

Aborigo, Raymond Akawire

Abouzahr, Carla

Adami, Hans-Olov

Afrifa-Yamoah, Ebenezer

Ahlström, Gerd

Ahmad, Riris Andono

Al-Khalisi, Nabil

Al-Naggar, Redhwan

Alam, Nurul

Alamoudi, Reem Mohammad

Allebeck, Peter

Allmark, Peter

Alvin, Marie Klingberg

Amugsi, Dickson Abanimi

Amuthan, Arul

Anh, Nguyen Quynh

Anthonj, Carmen

Arah, Onyebuchi

Arcaya, Mariana Clair

Ariani, Madelina

Armstrong, Susan Jennifer

Arteaga, Sergio Latorre

Aryeetey, Richmond

Asher, Laura

August, Furaha

Aurélie, Brunie

Awan, Furqan

Awasthi, Ashish

Baguma, David

Baig, Mukhtiar

Balcha, Taye Tolera

Bangha, Martin

Barnett, Adrian

Barten, Francoise

Bawah, Ayaga

Beran, David

Berezak, Nabil Slaoui

Berggren, Vanja

Bhandari, Tulsi Ram

Bhasin, Prerna

Bhatta, Dharma Nand

Bidwell, Posy

Bile, Khalif

Bird, Philippa

Bocoum, Fadima Yaya

Bodalal, Zuhir

Bonita, Ruth

Borg, Johan

Bosu, William

Bowen, Kathryn Jennifer

Boyce, Ross

Bozorgmehr, Kayvan

Brahmi, Fran

Buchta, William

Burström, Bo

Burton, William B

Caldwell, John A.

Caman, Ozge Karadag

Campbell, Irina

Cao, Guangwen

Chaiyakunapruk, Nathorn

Chandra, Urvashi

Chew, W.F.

Chhorvann, Chhea

Chola, Lumbwe

Choulagai, Bishnu P.

Chowdhury, Zafrullah

Christiani, Yodi

Chu, Kathryn

Churcher, Thomas

Claramita, Mora

Clark, Jocalyn

Cliff, Julie

Coe, Anna-Brit

Coetzee, Isabel

Colbert, Colleen

Combest-Friedman, Chelsea

Connell, John

Contreras-Urbina, Manuel

Cooper, Peter

Cordova Pozo, Kathya Lorena

Costa, Diogo

Dahlblom, Kjerstin

Danso, Nana Twum

Debeb, Hagos Godefay

Debelew, Gurmesa Tura

Dehghan, Mahlagha

Delisle, Hélène

Demont-Biaggi, Florian

Deskmukh, Pradeep

Dessie, Yadeta

Dewi, Fatwa Sari Tetra

Diaz, Carlos Alvarez-Dardet

Ditlopo, Prudence

Diwan, Vinod

Djalali, Ahmadreza

Doyal, Lesley

Drejza, Michalina Anna

Duclos, Vincent

Duffrin, Christopher

Dujardin, Florence

Duken, Eyasu Ejeta

Dykens, Jon Andrew

Ebi, Kristie

Ecclesfield, Nigel Edward

Eichbaum, Quentin

Elgammal, Hanan Abbas

Emilia, Ova

Engebretsen, Ingunn Marie S.

Enuameh, Yeetey Akpe

Farokhzadian, Jamileh

Faxelid, Elisabeth Anna

Fernbrant, Cecilia

Fiore, Louis David

Fontaine, Sherry

Ford, C.L.

Francis, Mark Rohit

Freij, Lennart

Ganatra, Bela

Garfield, Richard

Garre-Olmo, Josep

Geary, Rebecca Sally

Gebrehiwot, Tesfay Gebregzabher

Gebremariam, Mulugeta Betre

Geniets, Anne

Ghazinour, Seyedmehdi

Giné-Garriga, Ricard

Goldstein, Sue

Gomez-Olive, Francesc Xavier

Goodman, Annekathryn

Gouda, Hebe

Grollman, Chris

Gupta, Madhu

Guttmacher, Sally

Haafkens, Joke

Häggström, Christel

Hakimi, Mohammad

Hall, Charles

Hamilton, A.S.

Hamusse, Shallo Daba

Hanson, Lori

Haque, Aminul

Hardy, Lisa Jane

Hartjes, Laurie Beth

Hassan, Tayyab

Helmersson, Jing

Herbst, Abraham Jacobus

Hernandez, Alison

Hickman, Timothy

Hofman, Karen

Högberg, Ulf

Hollander, Anna-Clara

Hondras, Maria

Hörnell, Agneta

Hort, Krishna

Hounton, Sennen H.

Hu, Guoqing

Hunter, Ewan

Huo, Stephen Jinhai

Hussain-Alkhateeb, Laith

Ichoku, Hyacinth

Imam, Hisham Hussein

Iroezindu, Michael

Isaakidis, Petros

Isangula, Kahabi Ganka

Islam, Md. Saiful

Jaap, Angela

Jahn, Albrecht

Jat, Tej Ram

Jerdén, Lars

Jerling, Johann

Joshi, Pooran

Juvekar, Sanjay K.

Kaggwa, Esther B.

Kalengayi, Faustine Kyungu Nkulu

Kalita, Anuska

Kallestrup, Per

Kapiga, Saidi

Karar, Zunaid Ahsan

Karupaiah, Tilakavati

Kayser, L.

Kazlauskas, Evaldas

Khagayi, Sammy

Khanam, Masuma Akter

Kibria, Mohammad Golam

King, Carina

Kiriazova, Tetiana

Klipstein-Grobusch, Kerstin

Kohler, Stefan

Kohrt, Brandon

Koon, Adam

Kosen, Soewarta

Kowal, Paul

Krishnan, Anand

Ku, Grace Marie Verano

Kudale, Abhay Machindra

Kulkarni, Prashant Subhash

Kumar, Dewesh

Kushadiwijaya, Haripurnomo

Kusnanto, Hari

Kwaku, Daniel Azongo

Kyegombe, Nambusi

Laaser, Ulrich Rudolf

Lal, Dharmesh

Lazuardi, Lutfan

Lemard, Glendene

Leroy, Valeriane

Lessells, Richard John

Lestari, Septi Kurnia

Lewerin, Susanna Sternberg

Lindholm, Lars

Lindvall, Kristina

Lion, Casey

Litwin-Davies, Isabel

Löfgren, Curt

Lofters, A.K.

Loh, Alvona Zihui

Löhmus, Mare

Lokare, Laxmikant

López-Gatell, Hugo

Lucero-Prisno III, Don Eliseo III

Lundman, Berit

Lyons, Mary Winefride

Macfarlane, Sarah

Maclellan, Jennifer

Madiba, Sphiwe

Mahadik, Kalpana

Makwana, Sohil

Malangu, N.

Malik, Mohammad Umair

Malmusi, Davide

Målqvist, Mats

Mandal, Sema

Mangadan-Konath, Nabeel

Manthri, Suneedh

Marcos, Jorge

Mathur, Arvind

Mathur, Shiv Chandr

Mbenza, Benjamin Ben I Sasa Longo

Mcgetrick, Jennifer Ann

Mee, Paul

Mengistu, Mezgebu Yitayal

Merenda, Aluette

Mesias, Marta

Minh, Hoang Van

Mipando, Linda Alinane Nyondo

Mitchell, Carol

Mkoka, Dickson

Modh, Sandra

Moitra, Subhabrata

Monte, Dennis Decosto

Moodley, Jennifer

Morrell, Robert

Mumah, Joyce

Mundle, Robert

Muntaner, Carles

Mwashuma, Elizabeth Wanjala

Nakamura, Yasuhide

Nalugoda, Fred

Nalwadda, Gorette

Negrato, Carlos

Neuhann, Florian

Nguefack-Tsague, Georges

Nguyen, Quang Ngoc

Nguyen, Vi

Nisar, Yasir Bin

Nxumalo, Nonhlanhla Lynette

Nyary, Bryan

Obryan, Edward Conyers

Oh, Juhwan

Okike, Ifeanyichukwu O.

Oli, Natalia

Oliver, Martin

Onyango, Monica Adhiambo

Ortiz-Hernández, L.

Osaki, Keiko

Osooli, Mehdi

Ostergren, Per-Olof

Ozgur, Ozge

Palmieri, Patrick Albert

Patil, Shailaja S.

Paturas, James

Paul, Jake Robyn

Phalkey, Revati Keshao

Phillimore, Peter

Poluru, Ramesh

Portiño, Mercedes Carrasco

Prabandari, Yayi Suryo

Pradeepkumar, A.S.

Prashanth, N.S.

Pulkki-Brännström, Anni-Mari

Puthoopparambil, Soorej Jose

Puttkammer, Nancy H.

Quam, Mikkel Brandon

Ramokolo, Vundli

Rawal, Lal B.

Reddy, Srikanth

Rees, Kate

Reidpath, Daniel D.

Remes, Hanna

Renzaho, Andre

Ridde, Valery

Rispel, Laetitia Charmaine

Robertson, Jane

Rocköv, Joacim

Rohleder, Poul

Ruano, Ana Lorena

Rujumba, Joseph

Rutkow, Lainie

Sahlen, Klas-Göran

Salehi, Nasim

Salzmann-Erikson, Martin

Sankoh, Osman

Saunders, James E.

Schenck-Gustafsson, Karin

Schuftan, Claudio

Schuurman, Nadine

Senterre, Christelle

Seo, Mikyung Kelly

Sharma, Anjali

Sharmin, Sifat

Shiue, Ivy

Shore, William B.

Shukla, Sp

Silfverdal, Sven Arne

Singh, N.

Sjöström, Stefan

Sliem, Hamdy Ahmed

Smedman, Lars

Spigt, Mark

Srinivas, Sunita

Stebounova, Larissa

Steyn, Krisela

Stoll, Kathrin

Story, William T.

Subramanian, S.V.

Subramanian, Savitha

Sugumar, Raji

Sukums, Felix

Sun, X.

Suwannarong, Kanokwan

Swardt, Rina (Hc) De

Taegtmeyer, Miriam

Takahashi, Kenzo

Tampubolon, Gindo

Tizzoni, Michele

Tolhurst, Rachel

Towers, Andy

Travis, Lisa

Treves-Kagan, Sarah

Turinawe, Eb

Twine, Rhian

Tylleskär, Thorkild

Vaezghasemi, Masoud

Valentine, J.M.

Varghese, Beena

Vellakkal, Sukumar

Venters, Homer

Vilhelmsson, Andreas

Vio, Fernando

Visser, Maretha

Volken, Thomas

Vu, Kien Duy

Wagner, Ryan

Wai, Khin Thet

Wandera, Stephen Ojiambo

Wichro, Erika

Williams, Brian John

Williams, Carmel Jayne

Wilopo, Siswanto

Wiysonge, Charles Shey

Wojczewski, Silvia

Wolbring, Gregor

Wong, Teck Yee

Wyke, Sally

Xu, Biao

Yalcinkaya, Emre

Yawson, Alfred Edwin

Yaya, Issifou

Yuan, Beibei

Yüksel-Kaptanoglu, Ílknur

Zanchetta, Margareth

Zelenski, Yuri

Zhang, Jian

Zhang, Qi

Zimmer, Donald

Zulu, Joseph

